# Integrated Microfluidic Device for Enrichment and Identification of Circulating Tumor Cells from the Blood of Patients with Colorectal Cancer

**DOI:** 10.1155/2019/8945974

**Published:** 2019-07-01

**Authors:** Wentao Su, Hao Yu, Lei Jiang, Wenwen Chen, Hongjing Li, Jianhua Qin

**Affiliations:** ^1^Division of Biotechnology, Dalian Institute of Chemical Physics, Chinese Academy of Sciences, Dalian, China; ^2^University of Chinese Academy of Sciences, Beijing, China; ^3^First Affiliated Hospital of Dalian Medical University, Dalian, China; ^4^CAS Centre for Excellence in Brain Science and Intelligence Technology, Chinese Academy of Sciences, Shanghai, China; ^5^Institute for Stem Cell and Regeneration, Chinese Academy of Sciences, Beijing, China

## Abstract

Integrated device with high purity for circulating tumor cell (CTC) identification has been regarded as a key goal to make CTC analysis a “bench-to-bedside” technology. Here, we have developed a novel integrated microfluidic device that can enrich and identify the CTCs from the blood of patients with colorectal cancer. To enrich CTCs from whole blood, microfabricated trapping chambers were included in the miniaturized device, allowing for the isolation of tumor cells based on differences in size and deformability between tumor and normal blood cells. Microvalves were also introduced sequentially in the device, enabling automatic CTC enrichment as well as immunostaining reagent delivery. Under optimized conditions, the whole blood spiked with caco-2 cells passing through the microfluidic device after leukocyte depletion and approximately 73% of caco-2 cells were identified by epithelial cell adhesion molecule (EpCAM) staining. In clinical samples, CTCs were detectable from all patients with advanced colorectal cancer within 3 h. In contrast, the number of CTCs captured on the device from the blood of healthy donors was significantly lower than that from the patients, suggesting the utilization of the integrated device for further molecular analyses of CTCs.

## 1. Introduction

The spread of cancer, either by lymphatic drainage or distant metastasis through the peripheral bloodstream, could increase the death risk [1]. Although treated with surgical resection, approximately 20%–45% of colorectal cancer (CRC) patients developed local tumor recurrence or metastasis at distant sites [2]. Traditional serological tests offered limited information for early clinical symptom diagnosis and therapeutic response monitoring in a real-time manner. It is urgent to develop a reliable method to screen the early CRC patients and monitor antitumor response continuously [3].

Circulating tumor cells (CTCs), which are shed from the primary tumor and circulated in the bloodstream, may indicate the severity of metastatic progression. Identification, enumeration, and characterization of CTCs may provide a minimally invasive method for assessing the cancer status of patients and prescribing personalized anticancer therapy [4]. However, it is difficult to enrich CTCs from whole blood of patients, owing to their low quantity (about 1 CTC among ten million white blood cells and billions of red blood cells per milliliter) [5]. A variety of immuoaffinity-based approaches have been developed for enrichment of CTCs from peripheral blood, including immunomagnetic bead separation and flow cytometry [6–11]. For example, CellSearch™ system showed clinical validity regarding the monitoring of metastatic breast, prostate, and colon cancer [4, 5, 12, 13]. This approach relies on the enrichment of cancer cells from blood using EpCAM-coated magnetic nanoparticles combined with cell fixation and staining for visual CTC enumeration and identification. However, some invasive tumor cells may lose their EpCAM by an epithelial-mesenchymal transition (EMT) process [14, 15]. CTC enrichment based on targeting specific surface markers often leads to confused results and thus remains a point of controversy. Therefore, novel label-free technologies are desirable with a good precision for isolating CTCs from the circulated bloodstream of cancer patients.

Microfluidic technologies have come of age in the last 10–15 years and offer many advantages for the label-free separation and analysis of CTCs. Various microfluidic devices have been used to separate CTCs from a liquid biopsy. According to the physical property differences, these label-free techniques can be further divided into two subcategories: hydrophoresis (based on the cell size, density, shape, and deformability properties) [16–21] and dielectrophoresis (based on the cell dielectric property) [22, 23]. Among these technologies, the size- and deformability-based cell capture system is a commonly used label-free hydrophoresis technique because it is a relatively straightforward approach for cell separation mainly based on their size property. The size of microcavities is usually less than 10 *μ*m, and because of the larger size of tumor cells than red blood cells (RBCs), the blood cells can be filtered out while tumor cells are left behind. The pores of microcavity array can be also designed as many shapes, such as circular [24], oval [25], and rectangular [26]. However, these methods still lack the capabilities to realize CTC capture and analysis in a real-time or automatic manner.

In this report, we present the novel integrated microfluidic device for rapid isolation of CTCs with high purity in an automated manner. To isolate CTCs selectively based on size differences between CTCs and normal blood cells, multiple-cell trapping chambers with specific dimensions are fabricated on the microfluidic chip. Also, several microvalves are integrated to actuate the fluid flow, allowing to reduce manual operation procedures and integrate the CTC separation, staining, and detection processes. Spike-in tests and clinical tests were performed with the whole blood from healthy donors or patients to verify the practicability of the device for the isolation and detection of CTCs.

## 2. Materials and Methods

### 2.1. Microfluidic Device Fabrication

**Figure 1:**
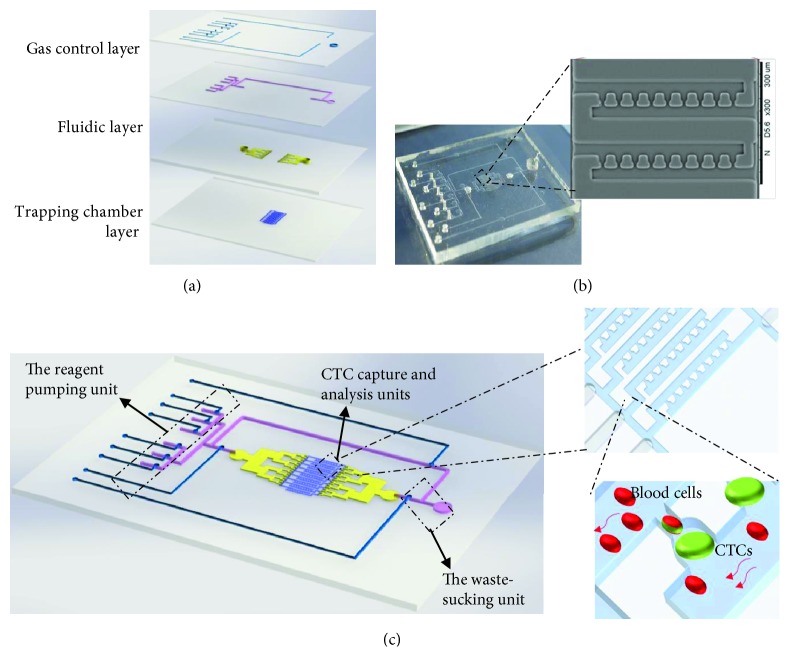
Schematic drawing of the integrated microfluidic device for CTC enrichment and analysis. (a) Layout of the integrated microfluidic device. It was composed of four layers, in which the top layer was a gas control layer containing microvalve and micropump channels. The two middle layers were fluidic microchannels, and the bottom layer contained microfeatures leading to multiple-cell trapping chambers with individual pore channels. (b) Photograph of the prototype microfluidic device. (c) A schematic representing how larger cancer cells got trapped in the trapping chamber while other blood cells with the smaller size escaped.

The multilayer CTC microfluidic device consists of four polymer layers as shown in Figure 1(a). The top layer is gas control layer that contains micropump channels and microvalves. The two middle layers are fluidic microchannels (~75 *μ*m deep), and the bottom layer contains multiple-cell trapping chambers (20 × 25 × 30 *μ*m) with separated pore channels. The corresponding master mold was designed by the AutoCAD software (Autodesk, USA) and prepared using soft photolithography techniques. SU-8 photoresist (Microlithography Chemical Co., USA) was spin coated onto clean glass wafer and then exposed to UV light (mask aligner UV-KUB-2, France) using the photomask described above. After removing the uncured photoresist, the glass wafer was put in a 180°C oven for 2 h to hard bake and be treated by chlorotrimethylsilane to reduce adhesion. The device were molded using a PDMS prepolymer (Dow Corning, USA) with a curing agent at 10 : 1 (*w*/*w*). The molded prepolymer was then cured by thermal curing at 80°C for 1 h and peeled off from the plates.

### 2.2. Cancer Cell Culture and Sample Preparation

Human colorectal carcinoma cell lines caco-2 (ATCC HTB-37) were used for spiked-in tests. The caco-2 were cultured in DMEM with 10% (*v*/*v*) fetal bovine serum (Gibco, USA) and 1% penicillin/streptomycin (HyClone, USA). The condition for the culture was maintained at 37°C with humidified atmosphere. The medium was changed every 48 h. When the cell lines reached 75 to 90% confluency, they were dissociated using 0.25% trypsin solution (Gibco, USA).

For sample preparation, peripheral blood samples from healthy donors or patients with colorectal cancer were received from the First Affiliated Hospital of Dalian Medical University. Samples were collected in Eppendorf tubes with ethylenediaminetetraacetic acid to prevent blood coagulation. 2 mL blood samples were collected from 7 colorectal cancer patients. For spike-in tests, a known amount of cancer cells were obtained using a microscope and dissociated by trypsin solution for each experiment. For the lysed blood samples, red blood cells were removed following the procedure of the red blood cell lysis kit (Beyotime Biotechnology, China), at a ratio of 10 mL lysis buffer to 1 mL of blood. Dissociated cells were directly added to 2 mL of PBS or lysed whole blood samples, which were loaded into the integrated device for CTC enrichment.

### 2.3. Device Operation for CTC Enrichment and Identification

Special buffer was pumped into the microfluidic device to remove bubbles before sample introduction, which consist of 1x PBS, ethylenediamine tetraacetic acid, and 0.5 % bovine serum albumin. Sample loading was conducted by applying pressure to the device through controlling the open or close of microvalves. The compressor and vacuum pump were used for controlling microvalves. In brief, the valves were closed by loading pressure (5 kPa) and opened by a vacuum (50 kPa) through the gas microchannels. The computer program was written by VC++ for the solenoid valve controlling [27]. CTCs with the bigger size were captured by trapping chambers, while other leukocytes with smaller size passed through pore channels and collected at the outlet reservoir. Larger leukocytes such as macrophages could be distinguished by their surface markers in the trapping chambers.

### 2.4. CTC Imaging and Analysis

For analysis of the captured cells, the immunostaining method was used. In order to identify and count the captured CTCs, CTC determination criteria EpCAM positive, CD45 negative, and DAPI positive were used. In each test, positive or negative controls were included for antibody staining and performance. Cells were incubated with a staining solution containing 4′,6-diamidino-2-phenylindole (DAPI) (Sigma-Aldrich, St. Louis, MO), EpCAM-FITC (VU-1D9, Abcam, UK), and CD45-PE (clone HI30, eBioscience, San Diego, CA). After immunostaining, the integrated device was monitored on a fluorescence inverted microscope (DMI 3000B, Leica, Germany). The enriched cells were enumerated and analyzed by the NIH ImageJ software. For scanning electron microscope (SEM) analysis of the microfluidic device, the surface of the capture chambers were coated with the Au layer (10 nm in thickness). The resulting samples were also conducted and imaged under 10 kV condition using SEM (HITACHI TM3000, Japan). The results were expressed as mean ± standard deviation (SD), and each experiment was performed in triplicate.

## 3. Results

### 3.1. Microfluidic Chip Design and Fabrication for CTC Capture and Analysis

In this study, we developed a novel integrated microfluidic device that can enrich and characterize CTCs in an automated manner. The design of the microfluidic device was shown in Figure 1(a), which composed of four layers. The top layer was a gas control layer containing microvalves and micropumps. The two middle layers were fluidic control layers with microchannels, and the bottom layer contains microfeatures leading to multiple-cell trapping chambers with individual pore channels. The photograph of the fabricated microfluidic device was shown in Figure 1(b) for size-selective CTC isolation and identification.

For CTC capture and analysis, several functional units were integrated on the single chip, including a CTC analysis unit, a reagent pumping unit, and a waste-sucking unit (Figure 1(c)). The CTC analysis unit had the function for CTC enrichment and identification. The unit consisted of about 5600 cell trapping chambers and a parallel network of individual pore microchannels (~10 × 8 *μ*m). The design of pore microchannel ensured that larger cancer cells got trapped in the trapping chamber while other blood cells with the smaller size escaped. Moreover, the single-cell trapping chamber integrated on the microfluidic device showed potential for downstream molecular analysis (e.g., PCR and FISH assays) at the single-cell level. The reagent pumping unit was designed for sample and immunostaining reagent loading. Six microvalves were designed to form micropump in the reagent channel, which delivered the required reagents from each reagent reservoir to the CTC analysis unit. The waste-sucking unit consisted with a microvalve in the waste channel, which was used for waste solution sucking. Using such chip design, the whole process for recovery, staining, washing, and detection of CTCs could be accomplished in an automated and simple fashion.

### 3.2. Microfluidic Operation for CTC Capture and Analysis

**Figure 2:**
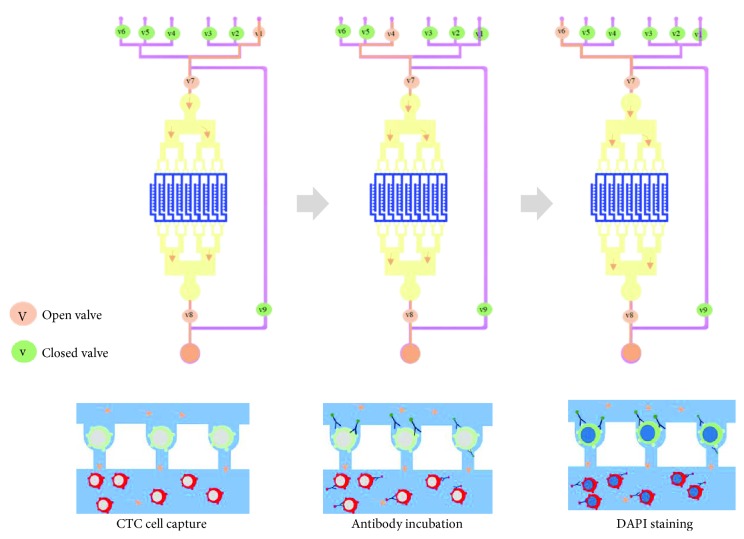
Schematic drawing of CTC enrichment and identification on the microfluidic device. The lysed samples, washing buffer (1x PBS), 4% PFA, blocking buffer, EpCAM-FITC, CD45-PE, and DAPI were loaded into the microchannel device in correct sequence by opening the microvalves in a simple manner.

For integrated CTC microfluidic capture and identification, the lysed samples, washing buffer (1x PBS), 4% PFA, 0.1% Triton X-100, blocking buffer, EpCAM-FITC, CD45-PE, and DAPI were loaded into the microchannel device in correct sequence by simply opening the microvalves (Figure 2). (a) The lysed samples were pumped from the sample reservoirs to the CTC analysis unit by valves 1, 7, and 8. Cancer cells with the bigger size were captured by trapping chambers. Sample loading time was 10 min at approximately 0.2 mL/min volumetric flow rate. (b) The PBS washing buffer was introduced into the CTC analysis units by valves 6, 7, and 8 to wash the trapping chambers for 5 min. (c) The 4% PFA solution was pumped into trapping chambers by valves 2, 7, and 8 to fix the captured cells for 10 min. Washing step was repeated. (d) The blocking solution with 5% goat serum (Life Technologies) was pumped into the trapping chambers by valves 3, 7, and 8 for 25 min. (e) Then, the monoclonal antibodies EpCAM-FITC and CD45-PE (1 : 100 dilution) were pumped into the trapping chambers by valves 4, 7, and 8 for 50 min. Washing step was repeated. (f) DAPI (Life Technologies) was finally pumped into the trapping chambers by valves 5, 7, and 8 for 5 min. After washing, the microfluidic device was ready for CTC identification.

### 3.3. Enumeration and Enrichment Efficiency of CTCs

**Figure 3:**
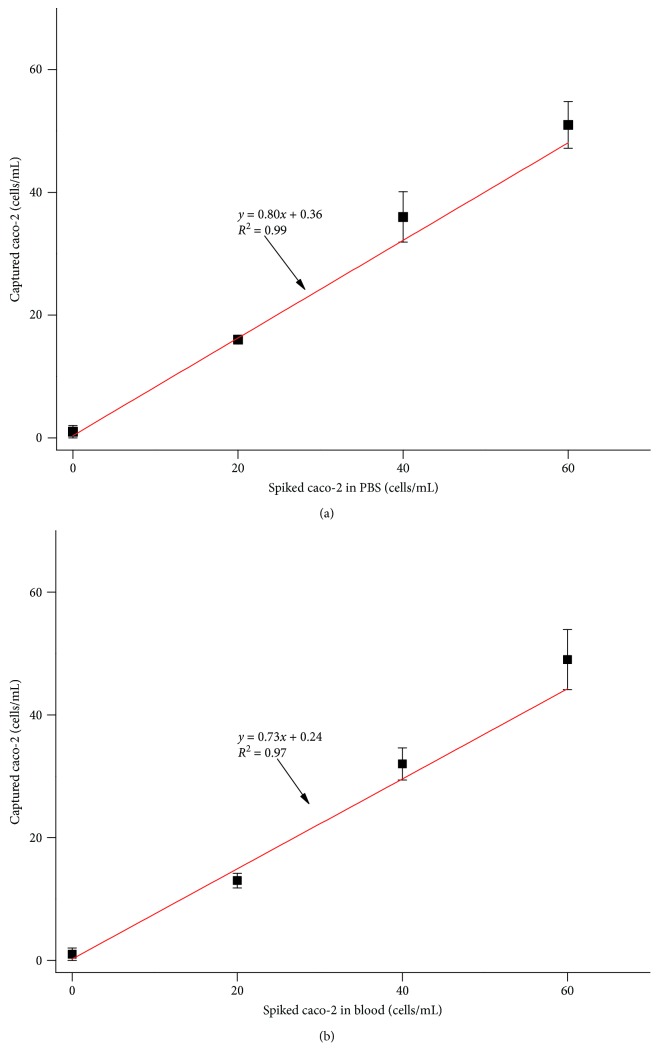
Capture efficiency of colorectal cancer lines spiked in PBS or the healthy donor blood. (a) The capture efficiency of cells using different cell lines in 1x PBS was used to show the performance of the device. (b) To assess cell capture efficiency under physiological conditions, a series of spike-in experiments in which a certain number of colorectal cancer were spiked into peripheral blood samples from healthy donors.

To examine the performance of the integrated microfluidic device, the samples of caco-2 cell lines which spiked in 1x PBS were firstly used for the CTC enrichment test (Figure 3(a)). Caco-2 cells were dyed with CellTracker CM-Dil fluorescent dye in the concentration ranging from 0 to 60 cells per 0.5 mL 1x PBS. The efficiency of cell enrichment ranged between 80 and 90% with average cell enrichment efficiency of 80%. The variation coefficient varied between 0.5 and 3.8 with three independent experiments (*n* = 3), suggesting high reproducibility of cell capture using this device.

To test the cell enrichment efficiency under physiological conditions, the samples of caco-2 cell lines which spiked into healthy peripheral blood were further conducted. As demonstrated in Figure 3(b), the cell capture efficiency in the spike-in samples ranging from 65 to 82% for caco-2 cells with the average cell capture efficiency of 73% depended on the amount of spiked cells. The result showed that the low variation coefficient varied from 1.2 to 4.9 with three independent experiments (*n* = 3). The results further demonstrated the high experimental reproducibility and enrichment efficiency using the integrated device, which were consistent with the results of spike-in experiment in PBS buffer.

### 3.4. CTC Analysis with Fluorescence Microscopy

**Figure 4:**
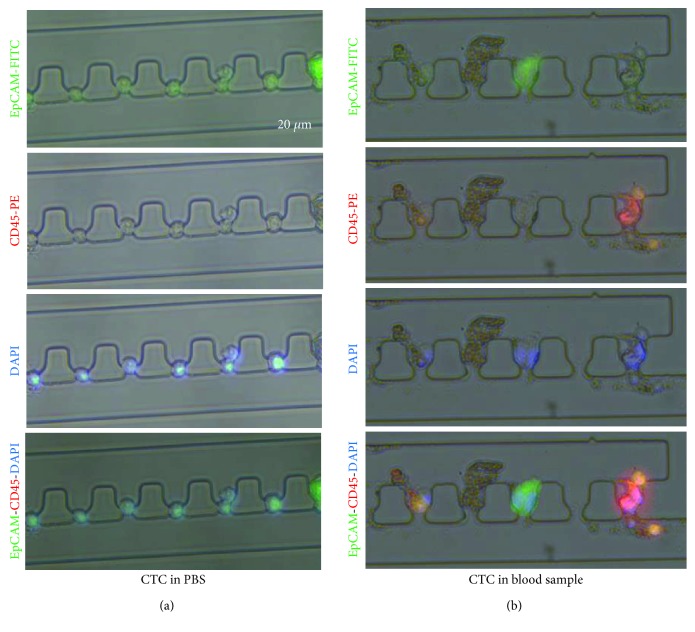
Immunostaining of captured cells in PBS or in human blood. A certain amount of caco-2 cell lines were first spiked into 2 mL (a) 1x PBS or (b) spiked into peripheral blood samples from healthy donors and then stained with either EpCAM-FITC or CD45-PE after being captured by microchambers. The cell nuclei were also stained by DAPI in all cases.

To further test the performance of the integrated microfluidic device, the enriched cells were characterized with fluorescence antibody staining. A series of immunostaining experiments were conducted to analyze the expression of colorectal cancer-specific biomarkers. A known amount of caco-2 cells were firstly spiked into 2 mL 1x PBS and introduced into the integrated device. The captured cells by the pore channels were then stained with EpCAM-FITC, CD45-PE, and DAPI (Figure 4(a)). The result revealed that caco-2 cell lines were positively stained with EpCAM antibody but negatively stained with CD45. Similar analysis had been performed using the spike-in samples into peripheral blood. The captured cancer cells and leucocytes were stained separately in Figure 4(b). CD45-PE was used as a marker for leukocyte staining to distinguish background leukocyte cells from the captured cancer cells. The results were highly consistent with those from the caco-2 with EpCAM positive and CD45 negative. The result suggested that the integrated microfluidic device was able to identify the differential phenotype of captured cells using specific biomarkers.

### 3.5. Enrichment of CTCs in Patient Clinical Samples

**Figure 5:**
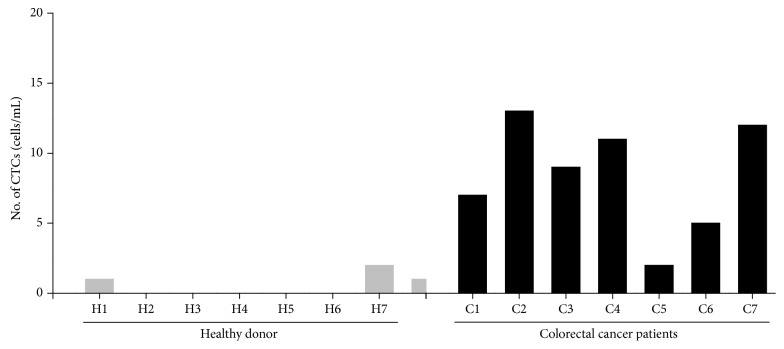
Results showing the performance of the CTC isolation microfluidic chip integrated with microvalves in colorectal cancer patient samples and healthy donors. The sample usage for CTC counts was normalized to 2 mL. CTC enumeration following antibody labeling was performed manually. EpCAM+/CD45-nucleated cells were identified as CTCs.

For clinical evaluation of the CTC isolation and analysis in the integrated microfluidic chip, 7 colorectal cancer patients and 7 healthy donors were enrolled in the study. For blood samples, 2 mL of whole blood was lysed by 20 mL of lysis buffer and directly pumped into the microfluidic device. The lysed blood sample loading as well as captured cell immunostaining were then conducted and generated in correct sequence by simply opening the microvalves. The data of actual CTC counts from colorectal cancer patients and healthy donors were provided in Figure 5. According to the results of immunofluorescence detection, CD45-positive hematologic cells (leukocytes) in majority of the blood samples were not captured by the device, which indicated that our integrated device showed a stronger specificity for CTC enrichment. Only 2 of the 7 healthy subjects in the control group had their cells detected by the device, and the number of detected cells was 1 and 1, respectively. These false positively stained cells were probably epithelial cells or leukocytes with EpCAM attached to the surface in whole blood [28]. In contrast, all the 7 tumor patients in the experimental group had their cells successfully detected. The actual number of isolated CTCs from colorectal cancer patients ranged from 2 to 13 CTCs. The results proved that the fabricated device could effectively detect CTCs in peripheral blood.

## 4. Discussion

In this study, we developed a novel integrated microfluidic device that can enrich and identify the CTCs from the blood of patients with colorectal cancer. This integrated microfluidic device had the ability for recovery, staining, washing, and detection of CTCs in an automated and simple fashion. Under optimized conditions, the enrichment efficiency for CTCs was greater than 73% in the cell-spiking experiment. In clinical study, CTCs were identified in all 7 patients with advanced colorectal cancer by epithelial cell adhesion molecule (EpCAM) staining. Thus, our device has potential as an efficient yet simple manner for fully automated CTC enrichment and identification.

Cancer metastasis and tumor recurrence are the main cause of cancer-related death, and dissemination of CTCs through the blood circulation is an important intermediate step [1, 8]. In contrast to invasive tissue biopsies that may impose a high risk to patients, CTC enrichment and analysis from circulating blood is considered as the real-time “liquid biopsy” in a noninvasive manner. CTCs show a great promise for potential clinical implications, including assessing prognosis of cancer and monitoring the therapeutic treatment, as well as serving as a surrogate biomarker for early diagnosis of cancer [8, 29, 30]. For those studies, the FDA-approved CellSearch system was regarded as the gold standard as the diagnostic tool, which worked for metastatic breast, prostate, and colon cancer. Despite its sensitivity, the CellSearch™ system highly relies on cell surface marker detection, which is not necessary, or low expression by CTCs [15]. Also, we should note that a population of CTCs may undergo the epithelial-mesenchymal transition (EMT) process to invade surrounding tissues and trigger distant metastasis [31]. In order to improve CTC detection sensitivity, it is essential to incorporate enrichment methods other than EpCAM-based ones.

Recently, several membrane filter devices are available for CTC enrichment based on the differential cellular size, including ScreenCell®, CellSieve™, and CellOptics [32–35]. Most of these filtering devices in common are provided solely for CTC capture, and the method and criteria for CTC recognition need to be established by researchers. On the other hand, our integrated microfluidic device covers all the steps from capturing the cells to staining them, resulting in much less manual effort and less turnaround time. The incorporated microvalves were used to realize the automatic CTC loading as well as immunostaining reagent delivery. Sample loading into the capture chambers and immunostaining of the captured cells on the microchannel device were then conducted and generated in correct sequence by simply opening the microvalves. Our microfluidics-based CTC detection platform may dramatically promote the current approaches for cancer diagnosis and prognosis. A further study would focus on a larger cohort to clarify the correlation between CTC count and clinicopathologic factors.

## Data Availability

The data used to support the findings of this study are available from the corresponding author upon request.
